# Sustaining Language Acquisition Research in Africa: A Commentary on Scaff et al. (2025)

**DOI:** 10.1111/desc.70063

**Published:** 2025-08-18

**Authors:** Paul Okyere Omane, Adebola A. Isaiah, Reginald Akuoko Duah, Thierry Nazzi

**Affiliations:** ^1^ CNRS‐Université Paris Cité Paris France; ^2^ Department of Linguistics and Nigerian Languages Kwara State University Ilorin Nigeria; ^3^ Department of Linguistics University of Ghana Legon Ghana

## 1 Introduction

The study by Scaff et al. (2025) provides additional evidence for the biases toward WEIRD populations in child language acquisition research. While their findings are unsurprising, they emphasize the urgent need to diversify research samples in terms of speaker population, geography, language, and culture (see also Aravena‐Bravo et al. 2023; Cristia et al. 2023; Kidd and Garcia 2022; Singh et al. 2023). Scaff and colleagues convincingly argue for greater diversity and generalizability in the field (see also Kidd and Garcia 2022; Singh et al. 2023), making a strong case for studying child language acquisition in more varied countries, more socioeconomically diverse populations (in terms of socioeconomic status [SES], education, and occupation), underrepresented rural communities, different family structures, and lesser‐studied languages from more diverse linguistic families and including more bilingual and multilingual infants with more varied language combinations.

Some of the issues raised by Scaff et al. (2025) can be addressed by researchers in WEIRD countries, for example, by including or targeting infants from lower SES families, and infants growing in rural areas of industrialized countries (e.g., Gonzalez‐Gomez et al. 2021), which will not be discussed here. Rather, in this commentary, we argue that studying language acquisition in rural and urban communities in Africa is essential for diversifying research on child language acquisition and addressing several of the other issues raised in the target article (i.e., language diversity; bi/multilingualism; sociocultural diversity), taking Ghana and Nigeria as examples. We also offer some suggestions to help create developmental corpora from underrepresented languages and speaker populations and, more generally, foster more inclusive language acquisition research.

Regarding language diversity, Scaff et al. (2025) report a dominance of Indo‐European languages, with English being the most prevalent among them. Although Africa is the most linguistically diverse continent, with approximately 2582 languages (Lodhi 1993), it is striking that not a single indigenous African language—particularly from the Niger‐Congo family, which is the largest with 1554 languages (Eberhard et al. 2025)—is represented in the languages or language combinations (for bilinguals and multilinguals) analyzed by Scaff and colleagues. This shows the extent of linguistic, geographical, and cultural biases in the data underlying most language acquisition theories. Corpus and experimental data from Ghana and Nigeria, where about 73 and 520 languages are spoken, respectively (Eberhard et al. 2025), could contribute to expanding diversity and our understanding of language acquisition processes and to fostering more generalizable theories. While efforts to build some corpora of endangered languages are currently being conducted (e.g., the Pangloss collection, which includes recordings of 258 corpora of underrepresented languages and dialects from 46 countries, https://pangloss.cnrs.fr/?lang = en; Adamou et al. 2025; Michailovsky et al. 2014), such efforts should be extended to language acquisition corpora and experimental research. Note that one difficulty in doing so is that for some of the world's languages spoken in small communities of less than a few thousand speakers, adults have stopped transmitting these languages to their children. Examples include the 14 Ghana‐Togo Mountain languages, namely Adele, Animere, Akebu, Ikposo, Lelemi, Igo, Tuwuli, Siwu, Sɛlɛɛ, Sɛkpɛle, Ikpana, Siya(sɛ), Nyagbo, and Tafi (Dakubu and Ford 1988; Agyekum 2010; Ameka 2017), spoken in southeastern Ghana and parts of Togo; and Kokomba, Tokoshi, Dindi, and Lukawa, all spoken in Kwara North, Nigeria (Lawal et al. 2016).

Second, Scaff and colleagues also report an overrepresentation of monolinguals in the CHILDES corpora. Again, studying language acquisition in Africa would help address this point, as children in many African contexts are raised in bilingual/multilingual homes and communities and are regularly exposed to multiple languages. This is true in both rural and urban areas, where multilingualism is a common aspect of daily life (e.g., Ghana: Nutakor and Amfo 2018; Nigeria: Isaiah 2023). For instance, in Accra (Ghana), infants between 3 and 12 months hear between 2 and 6 different languages in their environment, most of which are indigenous Ghanaian languages (Omane et al. 2025), while children aged 6 to 10 years and adults speak between 2 and 6 and 2 and 8 languages, respectively (Bodomo et al. 2010), suggesting the early and sustained multilingual experiences of children in Ghana. Similarly, in Nigeria, children often acquire two to three languages because of the environment in which they are raised (Bamgbose 1991). Moreover, the total number of language combinations (*n* = 22) in the bilingual corpora analyzed by Scaff and colleagues is fewer than the unique language combinations (*n* = 32) found in the multilingual sample of Omane et al. (2025), who studied Ghanaian infants in Accra. If we are serious about including more bilingual/multilingual children in our research samples, we must look beyond WEIRD contexts to underrepresented African settings.

Third, studying rural and urban African communities such as those found in Ghana and Nigeria could contribute to efforts to diversify the language acquisition field along cultural dimensions (Kidd and Garcia 2022), given the sociocultural environments in which infants grow up in these countries. In Ghana and Nigeria, caregiving practices and family structures expose children to a wide range of experiences, as they interact with multiple caregivers, including members of the nuclear family (mother, father, siblings), extended relatives (e.g., grandparents, aunts, uncles), and non‐relatives (e.g., co‐tenants, neighbors) (Igboanusi and Peter 2005; Isaiah 2023; Nutakor and Amfo 2018; Omane et al. 2025). These multi‐caregiver arrangements are closely tied to the types of housing in which children are raised. For example, in a compound house in Ghana—a structure that accommodates multiple households within a single compound—residents often include several nuclear and extended families who share common spaces such as playgrounds and cooking areas (Nutakor and Amfo 2018). In these communities, the socioeconomic status (SES) range is also varied. In many Ghanaian and Nigerian communities, especially in rural areas, language socialization is viewed as a communal responsibility, involving not just parents and older siblings but also extended relatives and community members. Children in these settings are cared for by various members of the compound, and our understanding of language acquisition would benefit from documenting this multi‐caregiver household structure, investigating how it may provide children with a form of socialization that may differ from what is experienced by children in populations that dominate current language acquisition research, and how it may impact language acquisition trajectories.

## 2 How (Or What Can We Do) to Get Corpora From Other Parts of the World

Developing a language acquisition science that is not Western‐centered and includes underrepresented communities and speaker populations is undoubtedly possible. We provide some steps that can help achieve this goal.

First, funding agencies must invest in this enterprise. Conducting language acquisition research involves a significant amount of work and also requires financial commitment. Given that researchers or local people in underrepresented communities in Africa may not have the financial capacity to undertake this important activity, we encourage funding agencies to support data collection and language acquisition research in these areas.

Second, language acquisition researchers must collaborate with experts in other disciplines, such as language documentation, ethnography, and sociolinguistics. This kind of cross‐disciplinary work can help foster experimental work and corpora creation in understudied communities. In addition, we also encourage collaboration between researchers from WEIRD and non‐Western contexts, as well as the local people. This could extend beyond an authorship role to include providing training to one another, which may involve data collection approaches in local communities and guidance on how to engage with community participants (e.g., a few cultural dos and don'ts; see Omane et al. 2023).

Third, we encourage researchers to provide international mentorship opportunities to students from underrepresented communities (see Aravena‐Bravo et al. 2023) who have the desire and passion to conduct research in language acquisition but lack access to experts in these fields at their local universities.

Fourth, encourage fieldwork. Most of what we know so far has primarily come from studies conducted in mainstream lab settings, predominantly in the WEIRD countries. Our efforts at diversification in naturalistic language research can benefit from undertaking fieldwork or expanding our research to underrepresented rural and urban communities.

Finally, based on our field‐based experimental research in Ghana and Nigeria, we have found that most community members have little to no knowledge of language development research. This lack of awareness significantly hinders participant recruitment and parents’ willingness to participate in our research. In our effort to promote inclusivity across cultures, populations, and languages, researchers working in underrepresented communities must provide meaningful feedback about their work. This is crucial, as the nature and impact of our research are often not immediately apparent to the communities we engage with.

In conclusion, as a field, our efforts to include language and population diversity will gain from focusing on children and languages in non‐WEIRD countries. Based on the examples of Ghana and Nigeria, with their rich linguistic diversity and in which bilingualism and multilingualism are the norm (typically not driven by immigration but rather being a lived, everyday reality), we argued that including underrepresented populations from Africa can help reduce biases, promote cross‐linguistic research, and support the creation of more diverse research samples, thereby fostering the generalizability of our research findings.

## Conflicts of Interest

The authors declare no conflicts of interest.

## Data Availability

Data sharing is not applicable to this article as no datasets were generated or analyzed during the current study.
